# CD4^+^ T Cell Depletion in Human Immunodeficiency Virus (HIV) Infection: Role of Apoptosis

**DOI:** 10.3390/v3050586

**Published:** 2011-05-12

**Authors:** Michèle Février, Karim Dorgham, Angelita Rebollo

**Affiliations:** 1 Unité Génomique Virale et Vaccination, CNRS URA 3015, Institut Pasteur, 28 rue du Dr Roux, 75015 Paris, France; 2 Inserm UMRS 945, Université Pierre et Marie Curie, Hôpital Pitié Salpêtrière, Bâtiment CERVI, 83 Bd de l‘hôpital, 75013 Paris, France

**Keywords:** apoptosis, HIV, viral proteins

## Abstract

Human immunodeficiency virus (HIV) infection is principally a mucosal disease and the gastrointestinal (GI) tract is the major site of HIV replication. Loss of CD4^+^ T cells and systemic immune hyperactivation are the hallmarks of HIV infection. The end of acute infection is associated with the emergence of specific CD4+ and CD8+ T cell responses and the establishment of a chronic phase of infection. Abnormal levels of immune activation and inflammation persist despite a low steady state level of viremia. Although the causes of persistent immune hyperactivation remain incompletely characterized, physiological alterations of gastrointestinal tract probably play a major role. Failure to restore Th17 cells in gut-associated lymphoid tissues (GALT) might impair the recovery of the gut mucosal barrier. This review discusses recent advances on understanding the contribution of CD4^+^ T cell depletion to HIV pathogenesis.

## 1. Introduction

Programmed cell death (PCD) and necrosis are two major processes by which cells die. Necrosis normally results from a severe cellular insult, which may lead to macrophage activation and the release of proinflammatory cytokines. These cytokines act as a “danger signal” and provide the costimulation required for T cell activation and immunity. Apoptosis, type I PCD, is a fundamental biological process in development and cellular homeostasis and occurs without inflammation or injury to surrounding tissues. Apoptosis is involved in embryonic development, hormonal regulation, immunity, regulation of inflammation and neoplasia. It is a controlled process, in organogenesis and tissue remodeling during development, to eliminate used-up, damaged, or misplaced cells during the embryonic development and the tissue homeostasis of multicellular organisms. Deregulation of apoptosis can disrupt the balance between proliferation and cell death. Failure of cells to die is an integral mechanism leading to malignancies such as cancer, and autoimmune phenomena. Conversely, an abnormal increase in cell death is observed in neurodegenerative disorders or in immunodeficiency [1]. So, in order to preserve homeostasis in a given system, it is crucial to maintain an equilibrium in the expression control among genes that promote proliferation, genes that influence survival/death and genes that inhibit proliferation.

**Figure 1:**
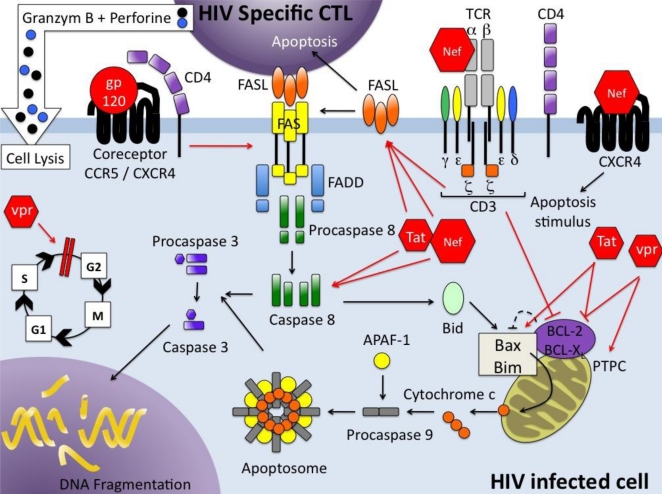
The extrinsic (death receptor-mediated) and intrinsic (mitochondria-mediated) pathways that lead to apoptosis of HIV infected helper T cells. The main HIV proteins (in red) implicated in CD4^+^ T cell death are the envelope glycoprotein gp120, the negative effector Nef, the transactivator of transcription (TAT) and the viral protein R (vpr). HIV gp120 uses CD4 on helper T cells and the chemokine receptors CCR5 or CXCR4 as coreceptors for virus cell entry and upregulates Fas-ligand (FASL) on these cells. Soluble Nef protein interacts directly with CXCR4 to induce cell apoptosis. Exogenous Nef protein directly stimulates TCR-CD3 complex and upregulates Fas/FasL expression on the cell surface while inhibiting the anti-apoptotic proteins Bcl-2 family. As endogenous Nef protein, Tat upregulates the Fas/FasL pathway and directly activates caspase 8. Tat and Vpr inhibit bcl-2 family while increasing mitochondria dysfunctions and cytochrome c release that promotes the formation of the apoptosome. HIV-1 Vpr also arrests cells in the G2 phase of the cell cycle. TCR: T cell receptor; CTL: cytotoxic T lymphocyte; FasL: Fas ligand; FADD: Fas-associated death domain; Caspase: cysteinyl aspartic acid-protease; BCL-2: B-cell lymphoma protein 2; BCL-X: BCL-2 like 1; Bax: BCL2 associated X protein; APAF: Apoptotic protease activating factor; PTPC : permeability transition pore complex.

There are a wide variety of stimuli and conditions, both physiological and pathological, which can trigger apoptosis. It is an energy-dependent process which implicates the activation of a group of cysteine proteases called caspases in a complex cascade of events. Depending on the initiating stimuli, two major mechanisms could be defined: the intrinsic pathway or mitochondrial pathway [2], that involves members of the bcl-2 family and mitochondrial functions, such as the role of mitochondrial membrane potential (Ψ_m_) in which fluctuations appear to be central to the distribution of proapoptotic molecules; and the extrinsic pathway which is activated by extracellular signals that act via death receptors (DR) [3,4] (Figure 1). Several DRs have been described that all belong to the tumor necrosis factor (TNF) receptor superfamily. Each pathway activates its own initiator caspase which, in turn, will activate the executioner caspase-3. There are evidences that the two pathways are linked and that molecules in one pathway can influence the other [5]. A third pathway, involving T-cell mediated cytotoxicity and perforin/granzyme dependent killing of cell works in a caspase-independent fashion (Figure 1). Autophagy, type II PCD, is a highly regulated physiological mechanism conserved among the evolution from yeast to mammals. Autophagy, a cellular catabolic pathway, is a process by which the cell, using membranes to isolate organelles or regions of cytoplasm, eliminates damaged organelles or consumes intracellular components as resources during starvation or other limiting condition [6]. It is a complex process as it can lead to cell survival as cell death. Apoptosis and autophagy are not mutually exclusive and the molecular regulators of both pathways are inter-connected leading to a cross-talk [7]. Autophagy is also a mechanism involved in innate and adaptive immunity against pathogens, and is a fundamental antiviral mechanism [8].

A fundamental characteristic of the immune system is its ability to expand rapidly the number of antigen-specific lymphocytes to combat pathogens. Apoptosis is of crucial importance for termination of the acquired immune response. This immune response is a multistep process: naïve T cells are activated through cross-linking of antigen to the T-cell receptor (TCR), leading to proliferation and differentiation into effector cells. After expansion of antigen-specific T cells, the majority of the activated T cells enter the so-called deletion phase and are eliminated to prevent undesirable immune responses such as autoimmunity whereas a portion of them survive as memory T cells [9]. The apoptotic process of elimination of activated T cells during the termination phase of an immune response is called activation-induced cell death (AICD) [10–12].

Pathogens have evolved molecules that affect the death pathway [13]. Human immunodeficiency virus (HIV) type-1 appears to induce cell death whereas other pathogens preserve their host by inhibiting the induction of a cell death pathway. This review presents an overview of the relationship between HIV-1 and CD4^+^ T cell death that complements the information presented in a recent review by Cummins and Bradley [14]. Literature concerning nonhuman primates naturally or experimentally infected with SIV (Simian Immunodeficiency Virus) is as important as literature concerning HIV-infected humans. This is the reason why we have decided to focus, in this review, only on recent data concerning humans infected with HIV-1, to try to understand how HIV is able to destroy CD4^+^ T cells which take a central position in the immune system.

## 2. HIV: Structure and Pathology

### 2.1. The Virus

HIV-1, the causative agent of AIDS (Acquired ImmunoDeficiency Syndrome) in human, belongs to the *Lentivirus* genus of the *Retroviridae* family. This family has a unique enzyme called reverse transcriptase that converts viral RNA to DNA upon viral entry into the cell. The genome of HIV, composed of two identical copies of single stranded RNA molecules, encodes nine open reading frames that produce 15 proteins, defining two general classes of proteins, structural and regulatory [15].

The three structural proteins are Gag, Pol and Env polyproteins which are subsequently proteolyzed into individual proteins: (1) the four Gag proteins, MA (matrix), CA (capsid), NC (nucleocapsid) and p6, (2) the two Env proteins, SU (surface or gp120) and TM (transmembrane or gp41) are structural components that make up the core of the virion and outer membrane envelope; and (3) the three Pol proteins, PR (protease), RT (reverse transcriptase) and IN (integrase), provide essential enzymatic functions and are also encapsulated within the particle. HIV encodes six additional proteins: Tat and Rev provide essential gene regulatory functions and four additional proteins often called accessory proteins, Vif, Vpr and Nef are found in the viral particle, whereas Vpu indirectly assists in assembly of the virion [16].

Cell entry of HIV is mediated by the binding of the viral envelope glycoprotein (Env) to the CD4 molecule on target cells along with a chemokine coreceptor, such as CCR5 or CXCR4 [17] determining the tropism of the virus (R5 or X4 respectively) for particular cell types (Figure 1). However, the majority of newly transmitted HIV strains uses CCR5 as a coreceptor [18]. CD4^+^ T cells, macrophages, monocytes and microglial cells are infected and killed by the virus [19,20], but activated CD4^+^ T cells are the optimal viral targets since HIV more efficiently infects and replicates in these cells [21]. After binding of the virus and entry, viral RNA is retro-transcribed and the provirus integrated into the cellular genome; virus proteins are then synthesized, the virus assembled and budding occurs.

### 2.2. Pathology

HIV is transmitted primarily through blood and genital fluids and to newborn infants from infected mothers.

HIV infection is principally a mucosal disease and events occurring in blood may not reflect events occurring at mucosal surfaces. The gastrointestinal (GI) tract is the major site of HIV replication, which results in massive depletion of lamina propria CD4^+^ T cells [22,23], in the first 3–6 weeks of infection and is maintained throughout the chronic phase. Years of antiretroviral therapy allow only a partial restoration of these CD4^+^ T cell populations. T cells in the blood or lymph nodes do not show the same degree of depletion: in acute HIV infection, blood CD4^+^ T cell numbers decline sharply, but as soon as antiviral immune response is established, these cells have the potential to reach a moderately subnormal level [24]. Following the initial peak of viremia, HIV-specific humoral and cell-mediated immune responses are readily detected; in particular HIV-specific CTL play a major role in the initial downregulation of virus replication in peripheral blood [25]. These immune responses generated by the host partly control viral replication, viremia declines by several orders of magnitude until it reaches a lower steady state level (viral setpoint); but these responses fail to eliminate the virus leading to a chronic infection in most individuals during an asymptomatic period which can go on several years. During the chronic phase of the infection, blood CD4^+^ T cell count declines slowly; this loss can be partially reversed by successful antiretroviral treatment, but it is accelerated during AIDS. Studies of large cohorts of HIV-infected individuals have clearly indicated that the rate of progression of HIV disease may be substantially different. Among the total population of HIV-infected individuals, the majority (70%–80%) belong to the group of typical progressors (the median time from initial infection to progression to AIDS is five to ten years). However, four additional subgroups have been identified [26]: rapid progressors who have an unusually rapid disease progression (AIDS or AIDS-related death within three years after seroconversion); the long-term nonprogressors (LTNP, two subgroups) [27] without progressive disease for several years (eight to ten) and the elite controllers with a viral load well below the detection limit of most conventional tests [28].

The viral persistence during the chronic phase of the infection is due in part to latent HIV reservoirs in resting CD4^+^ T cells [29] and additional cell populations [30,31]. HIV-1 infection causes a generalized state of immune dysfunction characterized by simultaneous chronic immune activation [32] associated with a paradoxical anergy in both CD4^+^ and CD8^+^ T cell compartments resulting in increased susceptibility to opportunistic infections and malignancy [33,34]. Proliferation of memory T cells is markedly elevated but the average lifespans of these cells are dramatically shortened [24,35].

## 3. Cellular Immunology of HIV

### 3.1. CD4^+^ T Cell Subpopulations

**Figure 2:**
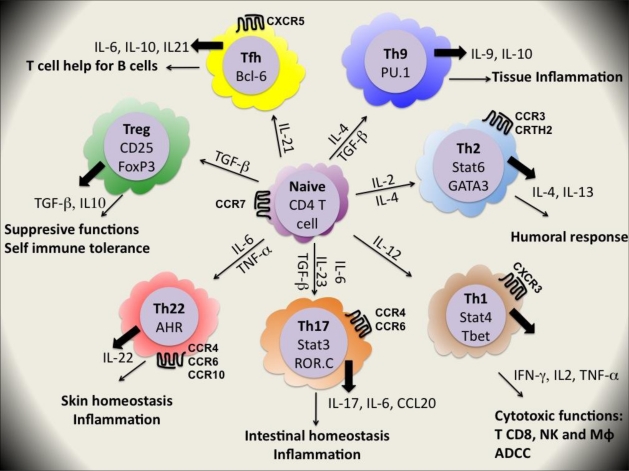
Differentiation of effector CD4^+^ T cells. Following activation by a given antigen, cytokines secreted in the microenvironment dictate the type of effector cells subsequently induced from naive T cells. Th1, Th2, Th9, Th17, TH22, T_FH_ and Treg lineages are defined depending on the expression of transcription factors, effector cytokines, and chemokine receptors. Fully functional Th cells contribute to the immune response.

CD4^+^ T cells orchestrate immune responses by differentiating into T helper (Th) cell subsets which recruit and activate other immune cells including B cells, CD8^+^ T cells, macrophages and other effector cells. The diverse functions of CD4^+^ T cells are determined by their cytokine secretion patterns and their tissue locations (Figure 2). In 1986, Mosmann *et al.* [36] divided T cell clones into two subsets, Th1 and Th2, which respectively produced the signature cytokines interferon (IFN)γ and interleukin (IL)-4 and IL-13. Th1 cells promote the cytotoxic effector functions of natural killer (NK) cells, CD8^+^ T cells and macrophages and are important for clearance of intracellular pathogens as viruses, intracellular bacteria and protozoan parasites. They also promote antibody-dependent cell-mediated cytotoxicity (ADCC) by supporting B cell production of IgG1 in humans. By contrast, Th2 cells promote humoral immunity, mediated by B cell-produced IgG4 and IgE in humans and are critical for clearance of extracellular parasites. The appropriate development of polarized Th cell responses to different classes of pathogens is still under investigation [37].

Most of CD4^+^ T cells reside within the gastrointestinal (GI) tract, lymph nodes (LNs) and other lymphatic tissues rather than in peripheral blood [38,39]. In the GI tract, the majority of CD4^+^ T cells are CCR5^+^ memory cells and constitute ideal viral targets [40,41]. Indeed these cells are very permissive to *in vitro* HIV infections [42].

Recently, this dualistic view of Th cell lineages has been complicated by the recognition of two new major subsets of Th cells, namely Th17 and Treg cells although others lineages may exist [43] (Figure 2).

Th17 cells, a subset of helper T cells, are identified *in situ* as a population of CD4^+^ memory T cells expressing the IL-23 receptor (IL-23R), CCR6 and the transcription factor ROR-γt. In peripheral blood, approximately 0.4% of CD4^+^ T cells are IL17^+^CD4^+^ T cells. Most of them are CD45RA^−^CD45RO^+^ [44]. They are important for intestinal homeostasis and are characterized by secretion of the proinflammatory cytokines IL-17, IL-1, IL-6, IL21, IL-22 and TNF-α. These cells arise exclusively from a population of CD161^+^CD4^+^ T cells in the presence of IL-1β and IL-23, and this precursor population has gut-homing potential (for review see [45]). They mediate inflammation and development of autoimmune diseases [46] but they also confer protection against extracellular bacteria, fungi and mycobacteria [47]. Th17 are involved in control of epithelial integrity of the gastrointestinal barrier and microbial invasion.

Regulatory T (Tregs) cells are a subset of circulating CD4^+^ T cells with suppressive properties implicated in the control of self immune tolerance [48], auto-immune diseases, cancer, transplantation, materno-fetal tolerance and inflammation induced by chronic pathogens [49,50]. They are phenotypically defined as CD4^+^CD25^+^FoxP3^+^; their development, maintenance and function require the expression of the master transcription factor FoxP3 (forkhead box P3) [51,52]. They express also the memory marker CD45RO and several activation markers such as HLA-DR [53,54]. Natural Tregs (nTreg) are generated in the thymus [55], would prevent autoimmunity and raise the activation threshold for all immune responses [56]. Adaptive or induced Tregs (iTregs) develop in the periphery from mainstream peripheral αβ T cells using self [57] or foreign antigens [58]. They are essential in mucosal immune tolerance and during normal homeostasis of the gut. Tregs have the capacity to actively block immune responses as they have been implicated in the suppression of T cell activation, proliferation and cytokine production through mechanisms not fully known [59]. They play a key role in regulating immune responses as a global “brake” on immunity [60]. Treg action is through the production of bioactive molecules, such as IL-10 and TGF-β as well as through cell-cell contact [61].

A lot of observations suggest flexibility in polarization of human cells. Memory T cells are considered flexible with regard to cytokine production. Several modes of plasticity of T cell subsets have recently been described (for review see [62]). T_FH_ (Follicular Helper T cells), Th3, Tr1 and Th9 have been proposed as new potential Th cell lineages but their formal status of subsets seems uncertain as they could represent subsets of Th1, Th2, Th17 or Treg lineage. Very recently, Th22 clones derived from patients with psoriasis and secreted IL22, and not IL-17, have been described. They infiltrate the epidermis in individuals with inflammatory skin disorders [63,64].

### 3.2. HIV and CD4^+^ T Cell Depletion

T lymphocyte numbers in the human body are kept constant by homeostatic mechanisms. These mechanisms fail in HIV infection characterized by progressive immune deficiency. Loss of CD4^+^ T cells and systemic immune activation are the hallmarks of HIV infection.

HIV pathogenesis can be divided into two major phases: the acute infection phase associated with a dramatic loss of CD4^+^ T cells residing in mucosal tissue, especially in GALT (gut-associated lymphoid tissue) [23,65] and a chronic phase characterized by an immune activation with a massive production of proinflammatory cytokines [66,67], which in turn is responsible for clonal deletion [32,68] and gradual loss of peripheral CD4^+^ T cells over time [68,69].

During primary HIV-1 infection, the number of CD4^+^ T cells declines in association with high viremia levels, before the onset of antiviral immune response. HIV-1 infects preferentially those CD4^+^ T cells that are HIV-1 specific, rather than CD4^+^ T cells specific for unrelated antigens [70]; moreover, *ex vivo* HIV-1 specific CD4^+^T cells have greater apoptotic potential than those specific for CMV [71].

Loss of CD4^+^ T cells after HIV infection is also a result of several mechanisms such as impairment of *de novo* production of T lymphocytes by the thymus, induction of syncytium formation, alteration of membrane permeability, mitochondrial dysfunction, killing by HIV-specific cytotoxic T cells or through expression of DRs due to heightened levels of immune activation. The importance of each will be discussed below. The thymus, the primary organ of thymopoiesis, is highly active during early life and thymic output and function progressively declines during ageing [72]. HIV infection leads to major alterations in T cell homeostasis due in part to destruction of thymic structures [73], reducing input of naïve CD4^+^ T cells into peripheral naïve T cell pool compared to uninfected individuals [74]. However, in almost all cases, loss of CD4^+^ T cells is associated with apoptosis which represents the major mechanism of CD4^+^ T cell depletion [75–77] and the number of apoptotic cells greatly exceeds the number of HIV-infected cells [77,78]. Apoptosis in lymph nodes is observed primarily in the HIV-negative cell fraction [79] leading to the conclusion that during HIV-1 infection, apoptosis occurs in bystander cells and not only in the productively infected cells themselves.

### 3.3. The Direct Cytopathic Effect of HIV-1

After HIV-1 infection, lymphoid tissue has been identified as a major site of HIV replication and a reservoir for HIV *in vivo* [80,81]. CD4^+^ T cells in the GI tract are 10-fold more frequently infected by the virus than are those in the peripheral blood [82], and the GI tract shows the most substantial CD4^+^ T cell depletion at all stage of HIV disease, which affects the CCR5^+^ CD4^+^ T cell subset, the majority of GI tract CD4^+^ T cells [22]. Infection frequencies of other mucosal lymphoid sites could be similar to those in blood (0.01–1% CD4^+^ T cells) [83].

The mechanisms of this cell death could have two explanations: a direct killing via virus-induced cytolysis by mechanisms related to direct infection of the cells [84] and the killing of virus-infected cells which occurs via the immune surveillance through the action of killer T cells [85,86]. HIV specific CD8^+^ T cells play a key role in the control of viral replication. Appearance of CTL responses at early stage of infection coincides with a rapid fall in plasma viremia [87]. CTLs recognize short epitopes associated with class I molecules of the major histocompatibility complex (MHC). However, selection of escape mutants is a major driving force of HIV evolution [88]. This phenomenon leads to an immediate decline of the corresponding CTL responses [89]. These responses exert a strong selection pressure, but as the founder epitopes are replaced by mutational variants, these responses are always race against the clock with *de novo* development of responses to epitope variants. Beneficial effects of CTL responses are largely impaired and do not avoid viral load at a high level during chronic infection [86].

Syncytia are generated by the fusion of HIV-infected cells, expressing Env (gp120/gp41) on the plasma membrane, with uninfected target, expressing a suitable coreceptor (CD4 or CCR5); however, the vast majority of syncytium-inducing HIV-1 variants employ CXCR4 as a coreceptor [90]. Syncytia are condemned to die by apoptosis after a latency phase explained in part by genomic instability [91], but p53 emerges also as a critical mediator of syncytial apoptosis [92]. Syncytia are frequently observed *in vitro* [93]. *In vivo*, biopsy and autopsy studies revealed that HIV-infected multinucleated cells, presumably formed by cell-cell fusion, are present in brain and lymphoid tissue of HIV-infected patients thus contributing to the depletion of CD4^+^ T cells. However, the overall extent of cell-cell fusion *in vivo* has not been estimated [93].

Whereas direct cytopathic effects affect the survival of infected CD4^+^ T cells, indirect mechanisms, such as activation-induced cell death, are likely to play a major role in elimination of uninfected CD4^+^ T cells, corresponding to “bystander” cells.

### 3.4. Hyperactive Immune State Upon HIV Infection

During chronic untreated HIV infection, practically every arm of the immune system that has been investigated has been shown to be in a hyperactive state: high T cell turnover, nonspecific T cell activation and proliferation, polyclonal activation of B cells and elevated proinflammatory cytokines are characteristic of HIV infection [94]. HIV, through the induction of immune activation, generates its own targets for replication.

A direct link between immune activation in chronic HIV infection and catastrophic events occurring at the mucosal surfaces during acute infection has been provided by recent studies. HIV causes a profound and complex disturbance of the mucosal immune function. In chronic HIV infection, intestinal permeability and enteropathy (diarrhea, gastrointestinal inflammation, malabsorption) are increased [95], a poorly controlled translocation of immunostimulatory microbial products occurs and correlates with immune activation markers, which in turn, are associated with disease progression [96].

Th17 cells are important for intestinal homeostasis [97]; they are involved in control of epithelial integrity of the gastrointestinal barrier and microbial invasion.

In healthy donors, there is a significantly higher frequency of IL-17-producing CD4^+^ T cells in the GI tract compared to peripheral blood (about 6% *versus* 2% respectively). During HIV infection, Th17 cells appear to be preferentially lost from the gastrointestinal tract, relatively early in the disease [98], even in patients with a high absolute CD4^+^ T cell count. In blood of chronically HIV-infected individuals, the proportion of Th17 cells is reduced 10-fold compared to HIV-uninfected controls [95].

Th17 cells represent ideal targets for HIV by virtue of high expression of the second receptor CCR5 and low secretion of CCR5 ligands MIP-1α and MIP-1β [99]. They are permissive to HIV infection *in vitro* and *in vivo*, but they do not appear to be the preferential targets of HIV [98]. Nevertheless, there is a preferential depletion of Th17 in the gut of HIV-infected humans. CD161^+^ CD4^+^ T cells, identified as gut homing Th17 precursor population [100], express particularly high level of CCR5, are permissive to HIV infection and are also lost during HIV infection. Depletion of Th17 cells and their precursors is mediated by direct infection of target cells, bystander apoptosis or a combination of mechanisms like other infected CD4^+^ T cells. Moreover, CD4^+^ T cells in HIV-infected patients are skewed toward a Th1 phenotype to the detriment of Th17 cells. This combined loss of Th17 and their precursor CD161^+^CD4^+^ T cells may contribute to impaired mucosal T cell immunity and microbial translocation [95]. The disturbed GALT function, depletion of Th17 and microbial translocation are accompanied by an incessant vicious circle of immune activation and inflammation with deleterious consequences on viral replication, T cell and epithelial death, and dysfunction of multiple additional cells [101]. One major consequence for the immune system is an increasing of activation-induced T cell death (AICD) leading to an exacerbation of physiologic mechanisms which control peripheral T cell depletion following an immune response [102].

A very recent paper revisits the mechanisms by which CD4 T cells, in lymphoid tissues, are depleted in HIV-infected hosts [103]. Authors suggested that the vast majority of bystander cell death in these tissues involved abortive HIV infection: naïve CD4^+^ T cells are refractory to productive HIV infection; after viral entry, infection is aborted as reverse transcription is initiated but fails to reach completion [104,105]. Accumulation of incomplete reverse transcripts in nonpermissive resting CD4^+^ T cells activates a host defense program that elicits proapoptotic and proinflammatory responses involving caspase-3 and caspase-1 activation.

### 3.5. HIV and Activation-Induced T Cell Death

During the termination phase of an immune response, the death of activated lymphocytes serves to limit the immune response by killing cells that are no longer needed. Molecular mechanisms involved in the death of peripheral T cells have been recently reviewed [12]. These mechanisms depend on the expression of TNF superfamily ligands and their receptors, e.g., Fas/FasL and TRAIL-DR5 [106,107,108] (Figure 1). After acute and during chronic HIV infection, immune activation is exacerbated and drives cells into apoptosis, reflecting an amplified normal process for homeostatic cell regulation.

Naïve T cells (CD45RA^+^) express little or no cell-surface FasL, while it is expressed in relatively large amounts by previously activated T cells (CD45RO^+^). Expression of c-myc is required for the activation-induced expression of FasL [109], upon which mature T-cell AICD depends. AICD utilizes at least in part the Fas/FasL system; but significantly, functionally distinct subsets of CD4^+^ T-helper cells have different sensitivities to AICD: after TCR ligation, Th1 cells express significantly higher levels of FasL and undergo AICD much more readily than do Th2 cells [110] (Figure 1). TRAIL contributes also to AICD in T cells but is exclusively observed in Th2 clones and primary T helper cells differentiated under the Th2 conditions [111,112]. Curiously, infected cells are more resistant to apoptosis than uninfected cells [113]; this involves a modulation of the mitochondrial pathway of apoptosis [114]. A consequence of this is that indirect cell killing via Fas/FasL will destroy activated but uninfected cells while sparing the fraction of infected cells.

Low frequencies of APC (CD13^+^ myelomonocytic cells, comprising macrophages, dendritic cells and granulocytes) were observed within the GI tracts of HIV-infected patients [98] and may contribute to an altered cytokine environment required for Th17 development and would favor the differentiation of CD4^+^ T cells along a Th1 rather than a Th17 pathway [115]. Th1 are highly sensitive to AICD and are lost more rapidly than the other Th cells [110]. Thus if Th17 cells, expressing the HIV receptor CCR5, and their precursors CD161^+^ T cells are lost mainly by cytopathic effect of the virus, and the cytokinic context in gut is modified by a change in proportion of antigen-presenting cells, favoring Th1 development instead of Th17, this could explain (in part) the preferential depletion of Th17 in the gut. As Th1 cells are very sensitive to AICD, in a context of hyperactive immune state, this could explain (also in part) the massive loss of CD4^+^ T cells during HIV infection.

### 3.6. Role of Regulatory T Cells in HIV Disease Progression

Tregs represent a heterologous population with different localizations but with equal suppressive capacities [54]. In healthy subjects, Tregs show higher turnover rates *in vivo* compared to conventional CD4^+^ T cells, without any immune activation [116,117]. The mechanisms of Treg cell function are still a matter of debate, but they can be grouped into four basic ‘modes of action’: suppression by inhibitory cytokines as IL-10 and TGF-β [118] suppression by cytolysis [119], suppression by metabolic disruption and suppression by modulation of dendritic cell (DC) maturation or function [120]. However, it seems that apoptosis induction in T effector cells is not important for human Treg mediated suppression [121].

Tregs, as conventional T cells, are progressively lost during HIV infection. Indeed, during HIV chronic infection, frequency of circulating Tregs is higher compared to normal controls, but their absolute counts are substantially decreased [122] and immune activation increases with decline in Treg count [95]. The fall in circulating Treg number may be explained by several mechanisms: preferential HIV infection and/or apoptotic properties of Tregs and/or relocalization in other lymphoid tissue.

Tregs express HIV co-receptors CCR5 and CXCR4, and are susceptible to HIV infection [53] only if they are previously stimulated [123]. Precursor population of Treg cells, termed naïve Tregs (nTregs), isolated from peripheral blood has been phenotyped as CD4^+^CD45RA^+^CD25^+^ and expressed high level of FoxP3 mRNA and protein. After TCR activation, these cells express high levels of HIV co-receptors CCR5 and CXCR4 and are preferentially infected by HIV early after activation, compared to naïve CD4^+^ T cells [124].

Little information is available regarding the homeostasis of Tregs and relevant mechanisms in chronic HIV infection. Treg cells display a rapid turnover level indicated by a proliferation marker (Ki-67) and apoptosis markers (active caspase-3 and Annexin-V) *ex vivo* in HIV-infected subjects. This turnover was associated with disease progression and is positively correlated with immune hyperactivation [125]. Freshly isolated human Tregs are highly sensitive to CD95-mediated apoptosis but show a relative resistance to AICD [126]. This susceptibility to apoptosis has also been attributed to low levels of the antiapoptotic molecule bcl-2 [116]. On the contrary, nTregs showing unique self-generating capacities seem to be more resistant to apoptosis [127].

Chronic HIV infection changes CD4^+^CD25^+^ Treg tissue distribution [128] with an increase of these cells in peripheral lymph nodes and mucosal lymphoid tissues where most HIV replication occurs: when frequencies of Tregs are compared in peripheral blood and in duodenal mucosa, the frequency and the absolute count of mucosal Tregs are highly increased in untreated HIV patients [129–131].

HIV binding on Tregs increases the expression of homing receptors CD62L and α4β7, enhances their homing to peripheral and mucosal lymph nodes, and enhances their survival [123]

HIV induces abnormal development of Tregs in the thymus, resulting in an enrichment of Tregs [74]. The consequences are ambiguous: as Tregs suppress T cell activation, they could be of either benefit—diminishing bystander apoptosis, T cell loss and hyperactivation—or detrimental—impairment of HIV-specific responses and participation to viral persistence. Study of natural hosts of SIV can yield important information regarding resistance to pathogenesis. After SIV infection, natural hosts (e.g., African green monkeys, sooty mangabeys) do not progress to clinical AIDS; they maintain high SIV viral loads, but avoid the chronic, generalized immune system activation associated with disease progression in HIV-infected individuals [132]. Both Th17 and Tregs are preserved. During HIV infection, frequency of Tregs is significantly increased in thymus, but input of these cells into peripheral T cell pool does not allow the preservation of this population as their absolute counts are substantially decreased [122]. The ability of this subset to elicit a beneficial effect is impaired.

## 4. HIV Protein and Apoptosis

Only 0.00001 to 0.01% of HIV-1 virions are infectious *in vitro* and *in vivo*. Thus, noninfectious virions may contribute to HIV-1 pathogenicity by inducing bystander T-cell apoptosis.

In addition to infecting and killing of CD4^+^ T cells, virtually every protein encoded by HIV can influence apoptosis in host cells [133] but the major players in HIV-induced apoptosis are Env, Nef, Tat, HIV protease and Vpr. They kill infected and uninfected lymphocytes through intrinsic or extrinsic pathways (Figure 1).

### 4.1. Env (gp 120)

Envelope glycoproteins have been implicated as the major cause of bystander cell death in T and other cell types [134]. Sources of gp120 are multiple: soluble gp120 resulting from shedding of the surfaces of the viral particles or infected cells, Env expressed on virions or at the surface of infected cells.

Cross-linking of the cellular receptor (CD4) and co-receptor CCR5 with gp120 activates the Fas/FasL (CD95/CD178) pathway and downmodulates a caspase inhibitor, the FLICE (FADD-like interleukin1β-converting enzyme)-like inhibitory protein (FLIP) [135]. This extrinsic pathway involves cell death receptors leading to the downregulation of bcl-2 and the activation of caspases 8 and 10, which in turn activate caspase 3 to initiate apoptosis. Intrinsic mechanisms of Env-mediated apoptosis have been described: engagement of CD4, expressed on uninfected cells, separately from TCR, with Env expressed on the surface of infected cells influences the expression of the proapoptotic protein Bax, which in turn induces dissipation of the mitochondrial transmembrane potential Ψ_m_ that could initiate apoptosis in lymphocytes [5]. CXCR4, a natural co-receptor of HIV Env, can also transduce a death signal when bound to Env through mitochondrial transmembrane depolarization, cytochrome C release and activation of caspase-9 [136].

Env from both R5 and X4 strains triggers autophagy and cell death in bystander CD4^+^ T cells [137]. It is a cell-type dependent process [138]. Currently, it is not known how autophagy is controlled, and what the importance of this phenomenon is in general CD4^+^ T cell depletion during HIV infection.

### 4.2. Nef

Nef (negative factor) protein is one of the earliest and most abundantly expressed viral proteins preliminary localized to cellular membrane. It is also present in the serum of infected individuals [139]. HIV-1 Nef protein (27 kDa) consists of four regions and its cellular localization depends on its conformation allowing interactions with many different cellular proteins. Among the pleiotropic effects of this protein, Nef modulates surface expression of various cellular proteins including CD4 and MHC class I and II, is required for the efficient replication of the virus and affects signal transduction pathways. Endogenous Nef upregulates both Fas and FasL [140]. Soluble extracellular Nef induces apoptosis in bystander T cells indirectly via the increased expression of FasL on infected cells [141] or directly by interacting with the CXCR4 chemokine receptor [142]. Nef can also be released in the plasma from HIV-infected individuals, in exosome-like microvesicles containing CD45 (leukocyte common antigen). As long as they contain Nef, these vesicles cause activation-induced cell death of resting CD4^+^ T cells [143,144]. The predominant mechanism of Nef-induced apoptosis is associated with death receptors; however, Nef may also trigger the intrinsic pathway by decreasing bcl-2 and bcl-XL expression and increasing caspase-mediated effects [145]. Depending on the situation, Nef can be anti-apoptotic [5,144].

### 4.3. Tat

Tat (Trans Activating Factor) is a regulatory protein of HIV indispensable for viral replication. Tat can be secreted in plasma from HIV-infected patients and can cross the cell membrane to enter uninfected cells [146,147]. This protein triggers extrinsic and intrinsic apoptosis pathways in both infected and uninfected cells [148]. For the first pathway, Tat induces the upregulation of Fas/FasL-mediated apoptosis, and when secreted it enhances the susceptibility of bystander cells to Fas-mediated killing [149,150]. Activated FOXO3a (Forkhead box transcription factor O class 3a) controls the expression of several proapoptotic genes, including FasL, Bim and TRAIL. Tat protein can activate the Egr1-PTEN-FOXO3a pathway leading to apoptosis of HIV-infected and non-infected cells [151]. Association of Tat with PTEN and PP2A promoters has been identified as the initiating event of Tat-mediated apoptosis [152]. Tat protein is implicated in the intrinsic apoptosis pathway through interactions with numerous intracellular targets. Tat stimulates the transcription of cyclin B1, which increases cyclin B1 level and promotes cell apoptosis [153]. Tat decreases bcl-2 [154] and increases Bax, caspase 8 expression [155]. Several mitochondrial interactions of this protein have been described: disruption of mitochondrial calcium homeostasis, down regulation of mitochondrial isoform of superoxide dismutase, translocation of Bim (Bcl-2 interacting mediator of cell death) from microtubules to mitochondria [5].

### 4.4. HIV Protease

The essential role of HIV protease is the cleavage of viral precursor proteins to yield mature virion proteins. In addition to its role in viral replication, HIV protease may also contribute to HIV pathogenesis. The protease substrate specificity is not restricted to viral proteins since the cytoprotective protein bcl-2 could be cleaved by HIV protease, leading to apoptosis [156]. Moreover, this viral enzyme directly cleaves procaspase 8 generating a novel peptide, casp8p41, and thus triggers the mitochondrial-dependent pathway of apoptosis that involves cleavage of Bid, loss of mitochondrial potential and nuclear fragmentation. After *in vitro* HIV-infection, almost all casp8p41^+^ cells are apoptotic whereas casp8p41^−^ cells are not. In cells from HIV-1 patients, this peptide is present only in CD4^+^ T cells, predominantly the memory subset, and initiates apoptotic cell death. Exogenous protease does not kill uninfected cells [157]. This mechanism may contribute to death of HIV-1 infected cells.

### 4.5. Vpr

Vpr (Virus protein R) is a virion-associated accessory protein necessary for virus replication [158]. It is expressed at the late stage of the virus life cycle, but is present during the early steps of infection because it is packaged into viral particles. HIV-1 Vpr exists in three forms: soluble, intracellular and virion-associated. Vpr, incorporated into the HIV-1 virion, shows multiple activities including nuclear transport of the preintegration complex to the nucleus, activation of the transcription, cell cycle arrest at the G2/M transition (cells infected with HIV-1 cease to proliferate) and induction of apoptosis. The cell cycle transition into G2 is required for Vpr to induce apoptosis. Muthumani *et al.* [159] demonstrated that Vpr induces apoptosis via the intrinsic pathway. Virion associated Vpr caused activation of initiator caspases 8 and 9 and effector caspases 3/7 and a drop of ψm confirming that death is initiated. Furthermore, the mitochondrial protein Bax (an independent mitochondrial pore-forming protein), has been identified as the key executioner of apoptosis in the context of HIV-1 *vpr* [160]. It is confirmed in human activated PBMCs [161]. It has been recently proposed that virion-associated Vpr could amplify Fas-induced cell death, a process that could involve the amplification effect of caspase 8 through the mitochondrial pathway [161]. So, Vpr can contribute to the depletion of CD4^+^ T lymphocytes either directly or by enhancing Fas-mediated apoptosis during acute HIV-1 infection.

## 5. Conclusion

HIV infection is associated with a progressive decline of circulating CD4^+^ T cells and loss of immune functions; however this infection shows a more severe depletion of CD4^+^ T cells in the gastro-intestinal tract than in blood [22,23,162]. In the acute phase of infection, the virus depletes CD4^+^ T cells in the mucosal tissue of the gut as they represent the “ideal targets” of the virus (activated CD4^+^ T cells, near the front door of the virus, at the lining of the vagina or anus). In this process, the virus also destroys the gut mucosas‘s structural cells, allowing gut bacteria or other pathogens to penetrate the body; these phenomena lead to irreversible damage to the immune system. Finally, HIV triggers chronic immune activation. Recently, a strong association between the destruction of intestinal CD4^+^ T cell homeostasis in the gut and the level of systemic CD4+ T cell activation [162] has been described.

The relationship between hyperimmune activation and loss of CD4^+^ T cells has been analyzed in different systems. First, natural hosts of SIV (HIV-like simian immunodeficiency virus), monkeys from Africa, do not show immune activation, do not lose their CD4^+^ T cells and do not evolve to AIDS whereas asian monkeys, nonnatural hosts, develop the pathology after SIV infection. It is fundamental to elucidate the mechanisms that allow natural hosts to coexist with SIV without overt disease [132]. Second, the initiation of antiretroviral therapy (ART) has significantly reduced morbidity and mortality of HIV-infected patients. This therapy has the ability to restore a normal circulating CD4^+^ cell count in most patients, associated with a low to undetectable plasma HIV RNA level [163]; however, there exists significant patient-to-patient heterogeneity as there is no consensus with regard to how to best define immunological success or failure of the treatment. From studies of large cohorts, up to 10 to 30% of patients fail to achieve CD4^+^ T cell counts of > 500 cells/μL and high levels of gut-associated HIV-DNA are associated with persistent immune activation and microbial translocation [164]. It has been hypothesized that this might be related to persistent dysregulation of gut CD4^+^ Th17 subsets [165].

Finally, the study of the immunological responses of resistant patients to the disease progression (LTNP and elite suppressors representing 10–15% and less than 1% of HIV-infected population, respectively) would give us important information concerning factors involved in disease progression and responses to be induced upon vaccination. Indeed, these populations are actively studied to understand how infected individuals control viral replication and immune activation for at least 10 years. Is it virus, or is it the host or is it all in the genes [166–171]? A very recent paper shows that LTNPs have intact CD4^+^ T cell populations in the gut mucosa with similar IL-17 expression and plasma LPS level to HIV-uninfected controls [172].

The next question is how can therapeutic strategies reproduce this privileged status without adapted genetic background? One thing appearing fundamental is the necessity to preserve the integrity of the gut mucosa; one approach would be to “test and treat”, a prevention strategy that promotes HIV testing and initiating antiretroviral therapy upon diagnosis, regardless of CD4 cell count [173]. According to the published results, the most important step to control HIV infection should be to manage the integrity of the gut mucosa.
